# Gastric Adenocarcinoma With Unusual Clear Tubulopapillary Morphology: A Case Report

**DOI:** 10.7759/cureus.53973

**Published:** 2024-02-10

**Authors:** Nadir Miry, Younesse Najioui, Anass Haloui, Nassira Karich, Amal Bennani

**Affiliations:** 1 Pathology Department, Faculty of Medicine and Pharmacy, Mohammed VI University Hospital, Oujda, MAR

**Keywords:** clear cell tubulopapillary adenocarcinoma, gastric papillary carcinoma, gastric clear cell adenocarcinoma, tubulopapillary adenocarcinoma, gastric adenocarcinoma

## Abstract

Gastric clear cell adenocarcinoma is an extremely rare variant of papillary adenocarcinoma of the stomach. It is associated with a poor prognosis due to its frequent lymphovascular invasion and also its higher risk of recurrence. It is characterized morphologically by a clear appearance of tumor cells, which can be easily confused with a metastasis of a clear cell carcinoma, particularly of renal origin. Very few cases have been previously reported in the literature, which makes it a very poorly known variant. Here, we report the case of a 64-year-old patient who presented with a polypoid lesion in the pylorus, revealed by epigastric pain and chronic vomiting. Histological analysis showed a gastric clear cell adenocarcinoma with a tubulopapillary architecture. Immunohistochemical examination excluded a metastasis of renal origin. Through this case report, we highlight the importance of recognizing such an uncommon and unusual variant of gastric adenocarcinoma, to prevent any potential misdiagnosis.

## Introduction

Clear cell adenocarcinoma is a rare and unusual variant of gastric papillary adenocarcinoma. Clear cell carcinoma is usually found in the kidney but can sometimes occur in the digestive tract and even less frequently in the stomach. To date, only a few cases of gastric clear cell papillary adenocarcinoma have been reported [1]. This type of adenocarcinoma is more commonly seen in the elderly population and often presents as a bulging or polypoid mass at the gastroesophageal junction. Histologically, gastric clear cell adenocarcinoma manifests with a papillary or tubulopapillary architecture. Tubules and papillae are lined by clear cells, which represent a variable proportion of the tumor [2-4].

Recognizing clear cell papillary carcinoma of the stomach is important in clinical practice. Distinguishing it from renal metastasis and other clear cell carcinomas of the stomach ensures accurate diagnosis and appropriate treatment strategies. This aids in improving prognostic outcomes and prevents potential misdiagnosis [5]. This variant is associated with a worse prognosis compared to the more conventional forms of gastric adenocarcinoma [3]. This highlights the importance of early detection and management, as well as the need for further research to enhance our comprehension and management of this rare type of gastric carcinoma.

## Case presentation

**Figure 1:**
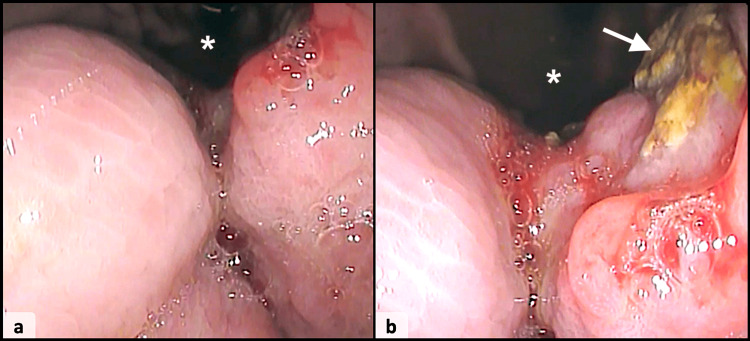
Endoscopic examination reveals a protruding and hemi-circumferential tumor of the gastric pylorus causing narrowing of the lumen (asterisk) (a), with an ulcerated surface (arrow) (b).

This is the case of a 64-year-old patient who presented with epigastric pain and vomiting for the past two months prior to consultation. The patient had no relevant medical history and did not report any other general symptoms. Physical examination revealed a tenderness in the epigastric region with no evident palpable mass. Endoscopic examination showed a polypoid mass measuring approximately 7×4.5 cm with a bulging and ulcerated surface located at the pyloric region (Figure 1).

**Figure 2:**
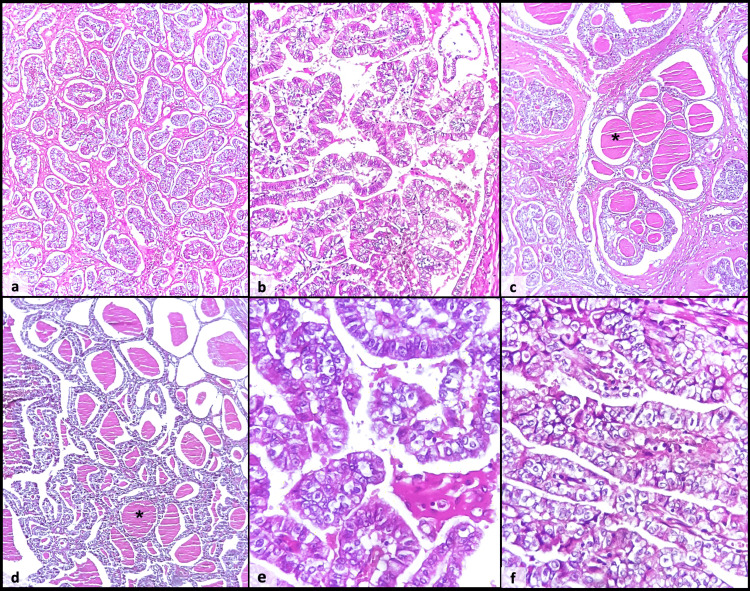
Microphotographs of the tumor showing multiple tubular and papillary structures, with well-developed fibrovascular cores (a and b). Tubular structures contain abundant intraluminal eosinophilic secretions (asterisk) (c and d). Tumor cells are cuboidal to columnar with evident cytonuclear atypia and a clear cytoplasm (e and f).

Multiple biopsies were performed. Histologically, the tumor is organized in multiple tubules and papillae, with evident fibrovascular cores (Figure 2a, 2b). The tumor cells are cuboidal to columnar with evident cytonuclear atypia presenting as enlarged, atypical nuclei, with fine chromatin and prominent nucleoli. The cytoplasm is abundant and often has a clear appearance (Figure 2e, 2f). Immunohistochemical analysis showed positivity of tumor cells for CK7 and partial positivity for cyclin D1 but no expression of CK20 and CD10, which was helpful in excluding a potential renal origin.

Additionally, the tumor shows abundant intraluminal eosinophilic secretions (Figure 2c, 2d, asterisk), which gave the tumor a remarkable morphological resemblance to thyroid carcinoma. Immunohistochemical staining for TTF1 and thyroglobulin was negative, allowing the exclusion of a thyroid origin. Therefore, the diagnosis of a clear cell papillary adenocarcinoma of the stomach was established.

## Discussion

Papillary adenocarcinoma of the stomach is a relatively rare variant, accounting for approximately 9% of all gastric carcinomas. It is defined by the 2019 WHO classification of digestive system tumors as a well-differentiated, exophytic tumor with elongated finger-like papillae made of a well-developed fibrovascular connective tissue lined by cuboidal to columnar cells [2]. The clear cell variant of papillary adenocarcinoma of the stomach is a poorly known subtype, which is notorious for its association with frequent liver metastasis and a low survival rate. Carcinomas with a clear appearance are rarely found in the stomach; they are mainly found in the kidney and female genital tract [3]. The clinical and histological characteristics of such variant are poorly known due to its rarity and the limited available data. This tumor mainly affects elderly individuals and appears grossly as a polypoid, exophytic mass, sometimes ulcerated, often located at the gastroesophageal junction [4]. In our case, the tumor was located beyond the gastroesophageal junction, specifically at the pyloric region. Clinically, it can be associated with a wide range of symptoms including abdominal pain, vomiting, or abdominal swelling [5].

Histologically, it presents with a papillary architecture [1]; cases of mixed tubular and papillary architecture have been described [4], as shown in our case. Clear cell carcinoma is defined, according to Kim et al., by the presence of a clear cell component representing at least 5% of the tumor. The clear appearance of tumor cells is linked to the intracytoplasmic accumulation of glycogen or mucin [3]. Immunohistochemical staining is important to rule out a metastatic lesion of a renal clear cell carcinoma; it shows overexpression of cyclin D1 and possible expression of alpha-fetoprotein (alpha-FP), glypican-3, and CD10 [1]. In our case, the absence of CD10 staining was useful in excluding a metastatic lesion of a clear cell renal cell carcinoma. Additionally, the presence of intraluminal eosinophilic secretions resembling those seen in thyroid carcinoma justified the utilization of thyroid-specific markers to eliminate the possibility of metastatic thyroid carcinoma.

Differentiating clear cell papillary adenocarcinoma from other clear cell carcinomas of the stomach, such as hepatoid carcinoma, adenocarcinoma with enteroblastic differentiation, and clear cell tubular adenocarcinoma, is crucial. Hepatoid adenocarcinoma exhibits a tubulopapillary architecture consisting of columnar neoplastic cells with clear cytoplasm, resembling early fetal gut. These tumors may express claudin-6, glypican-3, and alpha-FP [6]. On the other hand, hepatoid carcinoma presents with glandular structures with cuboidal clear cells and extensive geographic necrosis; it usually shows diffuse and strong expression of alpha-FP and glypican-3 [7]. Tubular adenocarcinoma is made of tubular structures with no papillae and tumor cells showing a variable degree of atypia [2]. In our case, the tumor is composed entirely of tubular and papillary structures, devoid of necrosis or any solid areas that could suggest an enteroblastic differentiation. Based on these morphological and immunohistochemical characteristics, the tumor was diagnosed as a clear cell tubulopapillary adenocarcinoma of the stomach.

The presence of a clear cell component is associated with a higher risk of lymph node metastases, vascular emboli, and a poorer prognosis, which can make treatment less efficient [3]. Thus, it can be more challenging for patients with this specific variant to respond to treatment, and patients may have a higher risk of recurrence or disease progression. Due to its rarity, there are no specific therapeutic recommendations, and its management generally follows the therapeutic protocols of classical gastric adenocarcinoma [1]. Through this case, we aim to emphasize the importance of recognizing such variants given their aggressive nature and frequent presentation in advanced stages of the disease.

## Conclusions

Clear cell papillary adenocarcinoma is a rare variant of papillary adenocarcinoma of the stomach. It is characterized by an unusual morphological appearance, often an admixture of tubules and papillary structures lined by atypical cells with clear cytoplasm. In this particular case, we highlight the importance of recognizing such a rare and unusual variant of gastric adenocarcinoma, due to its more aggressive outcome and unfavorable prognosis. Immunohistochemical study was essential in our case to rule out potential metastatic lesions in the stomach specifically of thyroid and renal origins.
